# Studies on phosphorus deficiency in the Qianbei-Pockmarked goat

**DOI:** 10.5713/ajas.18.0622

**Published:** 2019-01-02

**Authors:** Xiaoyun Shen, Yongkuan Chi, Bin Huo, Kangning Xiong

**Affiliations:** 1 School of Life Science and Engineering, Southwest University of Science and Technology, Mianyang 621010, China; 2 State Engineering Technology Institute for Karst Desertification Control, Guizhou Normal University, Guiyang 550025, China; 3 World Bank Poverty Alleviation Project Office in Guizhou, Southwest China, Guiyang 550004, China

**Keywords:** Qianbei-Pockmarked Goats, Phosphorus Deficiency, Calcium, Yunnan-Guizhou Plateau, Ruanguzheng Disorder

## Abstract

**Objective:**

Qianbei-Pockmarked goats are affected by a disorder locally referred to as ‘Ruanguzheng Disorder’, which is characterized by emaciation, lameness, muscular relaxation, stiffness of the extremities, and abnormal curvatures of the long bones. Our objective was to determine the relationship between the disorder and phosphorus deficiency.

**Methods:**

Tissue samples were collected from affected and healthy animals, while soil and herbage samples were collected from affected and healthy pastures. Biochemical parameters were determined using an automatic biochemical analyzer (OLYMPUS AU 640, Olympus Optical Co., Tokyo, Japan). Mineral contents in soil, forage, and tissue were determined using a Perkin-Elmer AAS5000 atomic absorption spectrophotometer (Perkin-Elmer, Norwalk, CT, USA).

**Results:**

The results showed that phosphorus contents in herbages from affected pastures were markedly lower than those from healthy areas (p<0.01), and the ratio of calcium to phosphorus in the affected herbages was 12.93:1. The phosphorus contents of wool, blood, tooth, and bone from affected animals were also markedly lower than those from healthy animals (p<0.01). Serum phosphorus values in affected animals were much lower than those in healthy animals, while serum alkaline phosphatase values from affected animals were markedly higher than those from healthy animals (p<0.01). Inorganic phosphorus values from affected animals were approximately half of that in the control group. Supplementation of disodium hydrogen phosphate prevented and cured the disorder.

**Conclusion:**

This study demonstrates that Ruanguzheng disorder in Qianbei-Pockmarked goats is primarily caused by phosphorus deficiencies in herbage due to fenced pastures and natural habitat fragmentation.

## INTRODUCTION

The Qianbei-Pockmarked goat is vital to the production system of the Yunnan-Guizhou Plateau in China. During the past five years, Qianbei-Pockmarked goats have been affected by a strange disorder known locally as ‘Ruanguzheng Disorder’, which is characterized by emaciation, lameness, muscular relaxation, enlargement of the costochondral junctions, stiffness of the extremities, and abnormal curvature of the long bones. Severe cases include permanent recumbency and eventual death.

The affected area is 26.7° to 27.5°N latitude, and 103.6° to 104.7°E longitude, at an average elevation of 2,600 m above sea level. Steppe and alpine meadows are the main vegetation types with affected goats [1]. Excellent autumn–winter ranges of native pastures were observed within the whole County until 2010 when the local government allocated pastures and stocks to individual families in an attempt to improve the local herdsman’s nomadic lifestyle and productivity. All animals grazed on the same pasture throughout the year, and as a result, Qianbei-Pockmarked goats developed Ruanguzheng disorder.

The disease has been observed throughout the years with a peak incidence occurring between July and October. In severe areas, epidemiological studies have indicated incidences of 28.33% in Qianbei-Pockmarked goats, which mainly occurred in mature females and lambs. Similar syndromes have been reported in cattle [2], water buffaloes [3], pigs [4], dogs [5], camels [6] and Guizhou semi-fine wool sheep [7], all of which were related to mineral deficiencies. However, no available information exists about the disorder affecting Qianbei-Pockmarked goats. Based on preliminary epidemiological and clinical observations, it was thought that Ruanguzheng disorder may also be a local nutritional and metabolic disease associated with mineral deficiencies.

The objective of this study was to determine the pathogeny of Ruanguzheng disorder and to establish the relationship between the disorder and mineral element deficiency.

## MATERIALS AND METHODS

### Ethics statement

Qianbei-Pockmarked goats used in this study were cared for as outlined in the Guide for the Care and Use of Animals in Agricultural Research and Teaching Consortium [8]. Sample collections in animals were approved by the Institute of Zoology, Chinese Academy of Sciences, Institutional Animal Care and Use Committee (Project A0066).

### Epidemiological investigations

Between April and November 2017, detailed investigations on the epidemiology of Ruanguzheng disorder were carried out in affected pastures. The data collected included ascertaining the history, incidence, character and regularity of the disorder, the natural ecological condition, and the effects on local animal husbandry. Data on the ecological and environmental conditions, and on the effects of diseases were obtained from local records and annual reports provided by the local government. Clinical signs were recorded by direct observation while following herds on the pastures. Upon clinical examination, body temperatures, pulse rates, respiratory rates, lymph nodes, and conjunctivae were normal. Upon further inspection, the extremities were flexed due to the relaxation of joint ligaments and the animals had an abnormal posture. Additionally, hooves were dry and back and animals had difficulties walking and standing. The weight was carried primarily on the heels.

### Sample collections

On August 1, 2017, ten herbage samples were collected from five affected pastures, and two samples from each pasture in an affected area in Xishui County, China. Ten herbage samples were also collected from healthy pastures in Zengyi County, China. To reduce soil contamination, forage samples were cut 1 cm above the ground [9]. At the same locations, twenty soil samples were taken from the surface layer (0 to 20 cm) using a 30 mm diameter cylindrical corer in affected and healthy areas. Each soil sample was composited by four soil cores collected at the site. Soil and herbage samples were dried at 60°C to 80°C for 48 h and passed through a 2 mm sieve.

On August 10, 2017, 20 Qianbei-Pockmarked goats with an average live weight of 21.65±1.31 kg, were selected for the study. Of the 20 goats, 10 goats aged 1 to 2 years showed symptoms of lameness and were picked from an affected area (multiple grazing animals in this area showed obvious clinical signs, including lameness, weakness, muscular relaxation, stiffness of the extremities, and abnormal curvatures in the long bones) in Xishui County of Guizhou Province, China. The other 10 goats, aged 1 to 2 years, were selected from healthy pastures in Zengyi County in Guizhou Province, where the disorder had not been reported. Clinical examination showed that all Qianbei-Pockmarked goats were in good health, and those animals were used as the control group.

Hair samples were taken from the neck of the Qianbei-Pockmarked goats, washed and degreased, and kept on silica gel in a desiccator until analysis [10]. Blood samples were obtained from the jugular vein using 1% sodium heparin as an anticoagulant, and stored at −4°C until analysis of mineral elements. Serum samples for biochemical analyses were separated by centrifugation (G: 10,000 to 15,000, time: 10 min, and plastic tube type: EF9976) and stored at −4°C in a vial [11].

After the experimental animal and the control animal were slaughtered, we conducted routine post-mortem pathological examinations by visually observing the tissues. Samples of hips, ribs and teeth were collected from animals to determine mineral elements. Humeri, femurs, and ribs were collected from a goat aged 1 year for pathological pictures, while radiographic images were obtained from the forelegs.

### Biochemical analysis

Serum electrophoretic studies of total protein, albumin, and globulin were performed using cellulose acetate [11]. Inorganic phosphorus (IP), lactate dehydrogenase (LDH), γ-glutamyl transferase, alkaline phosphatase (AKP), creatinine (CRT), and calcium (Ca) contents were determined using an automatic biochemical analyzer (OLYMPUS AU 640, Olympus Optical Co., Tokyo, Japan) [12]. Quality control serum was used to validate the blood biochemistry data (Shanghai Biochemical Co, China). All biochemical serum values were measured at 20°C [13].

### Analysis of mineral contents

Iron (Fe), copper (Cu), manganese, zinc, and Ca contents were determined using a Perkin-Elmer AAS5000 atomic absorption spectrophotometer (Perkin-Elmer, Norwalk, CT, USA) [12,14]. Molybdenum (Mo) contents were determined using flameless atomic absorption spectrophotometry (Perkin-Elmer 3030 graphite furnace with a Zeeman background correction) [6,11]. Fluorine was measured using ion chromatography (Metrohm MIC-7 Advanced, Herisau, Switzerland). Phosphorus was determined by spectrophotometry. The accuracy of the analytical values was verified by reference to certified values of elements in the National Bureau of Standards (bovine liver SRM 1577a) [6,15].

### Prevention and treatment

Twenty affected Qianbei-Pockmarked goats were selected from affected areas in Xishui County for a treatment experiment. Ten affected Qianbei-Pockmarked goats (two males, three lambs, and five females) were administered disodium hydrogen phosphate (Na_2_HPO_4_) orally at a dose of 60 g per Qianbei-Pockmarked goat and were allowed to graze in a fenced pasture. The treatment was repeated once per week from August to October 2017. The remaining affected Qianbei-Pockmarked goats grazed in the affected pasture without treatment. Clinical signs were recorded by direct observation of goat activities in the pasture.

### Statistical analyses

Data were analyzed using the statistical package for the social sciences (SPSS, version 20.0, Inc., Chicago, IL, USA), and presented as the mean±standard error. Significant differences between groups were assessed using the Student’s t-test with least significant differences of 1% (p<0.01) or 5% (p<0.05) [7].

## RESULTS

### Epidemiology

Disease mainly occurred in lambs and mature ewes throughout the year, with a peak incidence between July and October. Post-partum and pregnant ewes were most commonly affected by the disorder. Clinical symptoms were less obvious in mature males. In severe areas, 28.33% of Qianbei-Pockmarked goats were affected and mortality reached 47.14%. Aside from the symptoms described above, the long bones of affected Qianbei-Pockmarked goats were frequently broken without apparent stress. However, the respiratory rates, body temperatures, and heart rates of affected animals were healthy (Table 1).

### Autopsy findings

Visual autopsy examinations showed that the gross bone lesions of affected mature ewes and lambs were similar to those in affected Qianbei-Pockmarked goats. Almost all bones, particularly the scapula, mandible, ilium, ribs, and hip bones, were affected. The affected bones were brittle, porous, light, susceptible to fracturing, and easily cut and sawn. The marrow cavity was enlarged and extended into the epiphysis, and the cortex was spongy, thin, and soft. Spontaneous fractures frequently occurred on the pelvises and ribs of affected Qianbei-Pockmarked goats.

**Figure 1:**
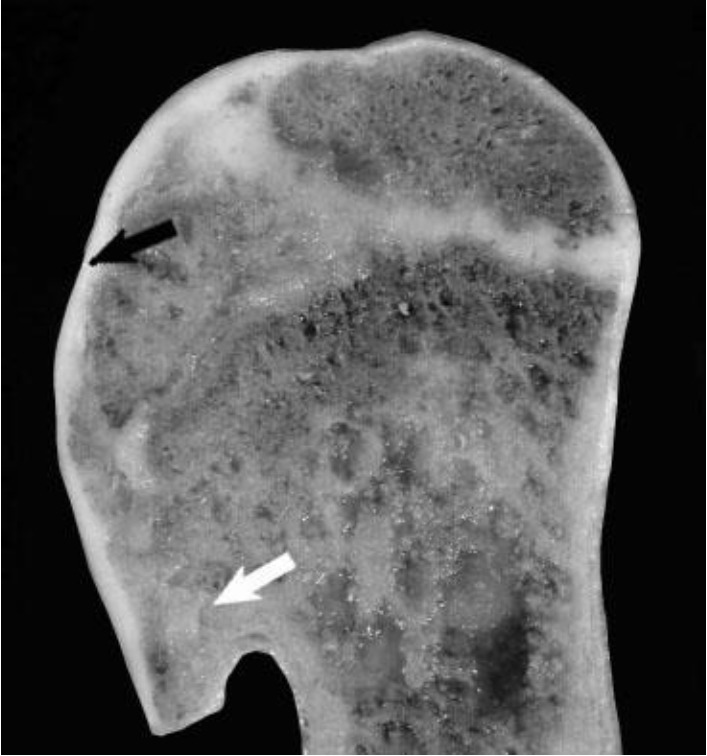
Proximal humerus image of 1-year-old Qianbei-Pockmarked goat with phosphorus deficiency, showing a flattening of the humeral head and separation of articular cartilage from collapsed subchondral bone (black arrow). Also shown is segmental thickening of the physis (white arrow), thickened metaphyseal trabeculae, and thickened cortices.

**Figure 2:**
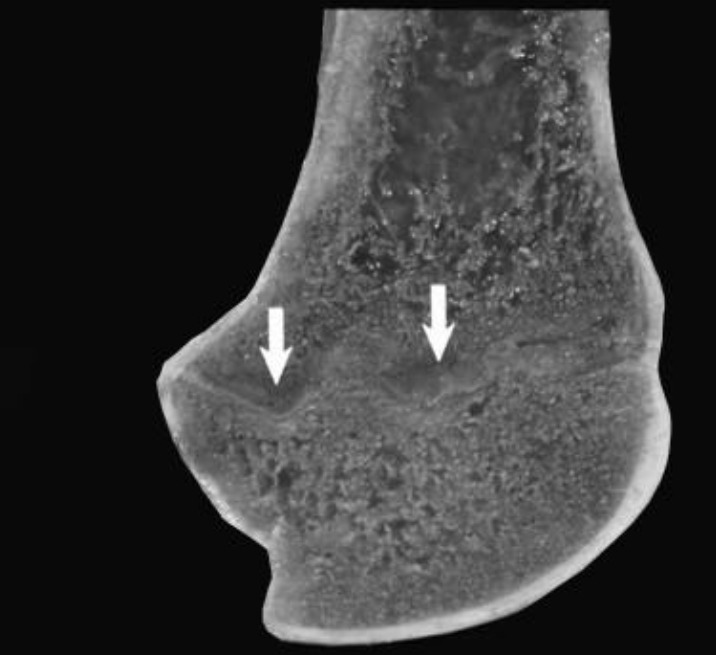
Distal femur from a 1-year-old Qianbei-Pockmarked goat with phosphorus deficiency. White arrows indicate segmental physeal thickening.

**Figure 3:**
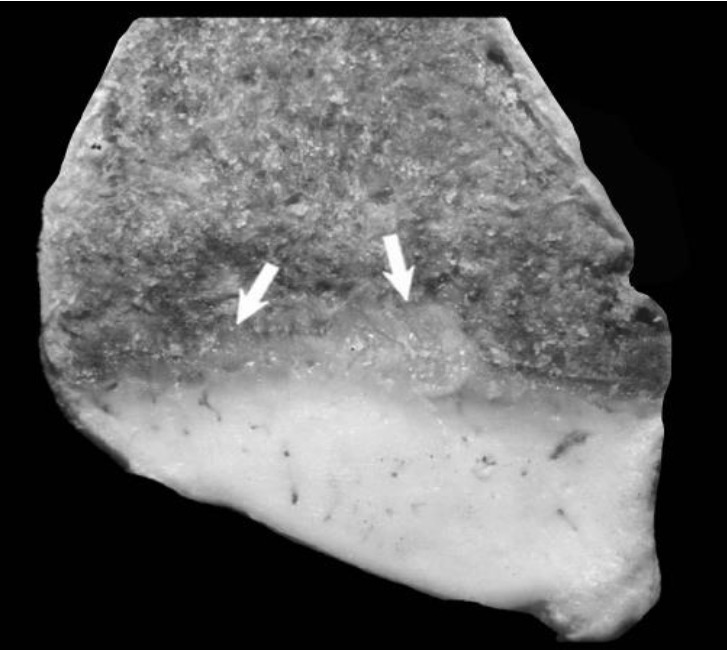
Costochondral junction from a 1-year-old Qianbei-Pockmarked goat with phosphorus deficiency. White arrows indicate tongues of cartilage extending into the metaphysis of a costochondral junction.

**Figure 4:**
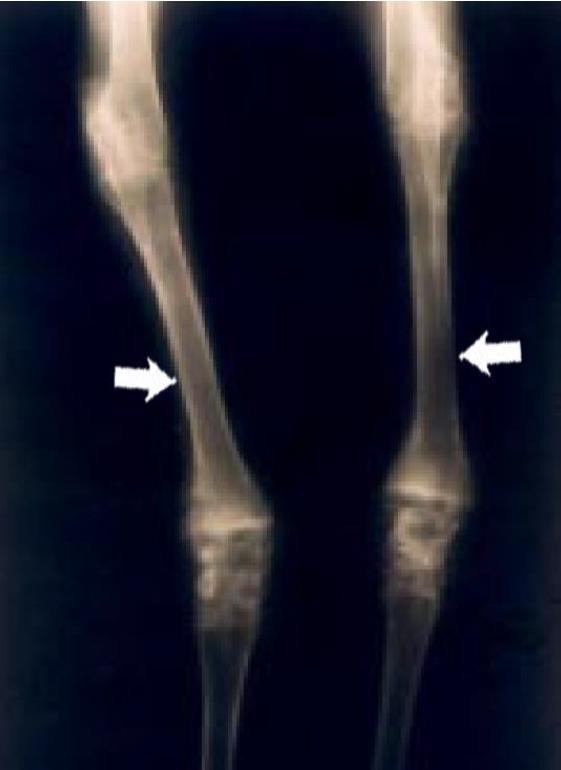
Radiographic view of the forelegs of a 1-year-old Qianbei-Pockmarked goat with phosphorus deficiency. The medullary canals were wide and the cortex was thin in the long bones of the forelegs (White arrows).

Proximal humeri showed flattening of the humeral head and separation of articular cartilage from the collapsed subchondral bone (Figure 1). Segmental thickening of the physes, thickened metaphyseal trabeculae, and thickened cortices in proximal humeri were also observed (Figure 1). Distal femura showed segmental physeal thickening (Figure 2). Impaired provisional calcification of cartilage at sites of endochondral ossification led to the accumulation of hypertrophic chondrocytes, resulting in thickened and irregular growth plates with islands and tongues of chondrocytes extending into the metaphyses (Figure 3). The enlargement of joints with an apparent bowing of the long bones and a broadening of the epiphyses were also typical. Irregular ulcers were also observed on the surface of joints in affected Qianbei-Pockmarked goats (Figure 4). Lesions were most severe in the fastest-growing bones, including the radius, tibia, metacarpals, and metatarsals. Radiographic analyses revealed that widening of the physeal growth plates was the most archetypal change. Other abnormalities observed upon radiographic analysis included metaphyseal flaring, thinning of the cortex, poor mineralization of the skeleton, and pathological fractures (Figure 4). Similar changes occurred beneath the articular epiphyseal cartilage complexes in the expanding epiphyses of young goats. Other microscopic changes included the development of thick osteoid seams lining trabeculae and the disorganization or absence of the primary spongiosa. Hemorrhage and signs of trauma were observed in the metaphyses and primary spongiosa due to damage to the weakened trabeculae of poorly mineralized bone.

### Biochemical results

Serum levels of CRT, LDH, and AKP from affected Qianbei-Pockmarked goats were markedly higher than those in the healthy goats (p<0.01), while IP values were approximately half of those in the control group. The AKP levels of affected Qianbei-Pockmarked goats were approximately two times higher than that of healthy goats (Table 2). Similarly, the levels of serum α-globulin and β-globulin in affected Qianbei-Pockmarked goats were markedly higher than those of the control group (p<0.01). Serum levels of γ-globulin in affected Qianbei-Pockmarked goats were markedly lower than those in the control group (p<0.01) (Table 3). There were no differences in other biochemical values between healthy and affected Qianbei- Pockmarked goats.

### Mineral elements

Phosphorus contents of the herbage and soil in the affected pastures were markedly lower than those in the healthy pastures (p<0.01) (Table 4). Phosphorus contents in the herbage in healthy pastures were 5.56 times higher than those in the affected area. The Ca:P ratio in herbage of the affected pastures was approximately 13:1. Other mineral contents were within the healthy ranges. Phosphorus contents in wool and blood samples and in hips, ribs, and teeth from affected Qianbei-Pockmarked goats were approximately half of those in healthy animals (Tables 5, 6).

### Treatment and prevention

Affected Qianbei-Pockmarked goats treated with dibasic sodium phosphate (Na_2_HPO_4_) recovered gradually within 15 to 20 d. Appetites improved rapidly and the signs of lameness that were observed in most affected Qianbei-Pockmarked goats improved within 5 to 15 d following treatment. However, foreleg deformations recovered slowly and required prolonged treatments. Lambs and mature ewes were more vulnerable than treated and healthy male Qianbei-Pockmarked goats. Ten treated Qianbei-Pockmarked goats survived. Among the 10 untreated Qianbei-Pockmarked goats, two lambs and three mature females survived, while two females and three lambs eventually died.

The phosphorus contents in blood were significantly increased in the treatment group (p<0.01), and reached a healthy value at 10 day. The same result was not observed in the control group. No significant changes in the concentration of other elements were observed in the blood of the treatment group (Table 7).

## DISCUSSION

Specific minerals have been related to disorders in livestock and wildlife in the literature. Huang et al [16] reported the pathogenesis of Tibetan sheep and goats due to sulfur (S) and Cu deficiencies in forage in Gansu province, China [14]. Shen et al [15] reported on a disease of semi-fine wool sheep in Guizhou province that was related to a sulfur deficiency caused by high Fe in forage. The main signs of the disease included wool-eating, emaciation, loss of appetite, pica, and weight loss [11]. Yuan et al [13] reported another disease of semi-fine wool sheep in Guizhou Province that was caused by Cu deficiencies due to high S and Mo contents in forage. Burk et al [17] reported on a disorder of some patients to selenium deficiencies. The main signs of such disorders included a loss of appetite, emaciation, pica, anemia, necrosis of skeletal muscle, and weight loss. Compared to that disorder, the disorder presented here occurred in Yunnan province, which is adjacent to Guizhou province, and has different characteristics and mineral element deficiencies. This was the first report of Ruanguzheng disorder of Qianbei-Pockmarked goats.

Clinical and epidemiological observations indicated that Qianbei-Pockmarked goats suffered a mineral metabolic disorder associated with phosphorus deficiencies. Such studies revealed that the phosphorus contents in the soil and forage in the affected pastures were markedly lower than those in healthy areas. In addition, phosphorus values in serum, bones, wool, and teeth from affected Qianbei-Pockmarked goats were significantly lower, while serum AKP values were markedly higher than those of healthy Qianbei-Pockmarked goats. The results were consistent with the response criteria in phosphorus deficiency disorders of camels, sheep, and yaks [6,12,18,19].

Oral supplement of dibasic sodium phosphate appeared to cure the disorder. Appetites improved rapidly and signs of lameness in most animals improved within 5 to 15 d following treatment. However, foreleg deformations recovered slowly and required prolonged treatments. Lambs and mature ewes were more vulnerable than males in the treated and healthy Qianbei-Pockmarked goats. Among the 10 untreated animals, two lambs and three mature females survived, while two females and three lambs eventually died. Such results demonstrate that the disorder is related to phosphorus deficiencies in herbage, which is attributable to current herding practices.

The local herding practices have a great impact on the mineral element imbalances in grazing livestock and wildlife in China [20,21]. In the 2000s, pastures and livestock were allocated to individual families. As a result, the fenced pastures may have created mineral element imbalance disorders in animals.

Mineral contents in soil and herbage are spatially distributed [22]. If animals graze in an extensive area, they have the opportunity to graze in pastures with rich and poor nutrition. Therefore, mineral deficiencies in grazing animals is minimal [23]. In this study, the phosphorus contents of soil and herbage from affected areas were markedly lower than those in healthy pastures. Qianbei-Pockmarked goats foraged in the same pastures with phosphorus deficiencies throughout the year. As a result, Qianbei-Pockmarked goats suffered from a disorder related to phosphorus deficiency.

For many grass species, the grazing periods with relatively high available phosphorus contents (>0.3%) are short [4,9,24]. In most years, mature herbage contains P <0.15% [25,26]. In general, sufficient phosphorus contents for ruminants are >0.005% in soil and >0.3% in herbage [23,24]. In this study, phosphorus contents of the soil and herbage of affected pastures were 0.0038% and 0.022%, respectively, which were much lower than acceptable levels.

A mean Ca:P ratio of 1:1 to 2:1 is recommended for proper utilization of minerals by livestock [27,28]. Dietary Ca:P ratios <1:1 or >7:1 adversely affect growth and feed efficiencies [29]. In the present study, the Ca:P ratio in forage from healthy areas was 2:1. However, the Ca:P ratio in herbage from affected pastures was approximately 13:1, which had a negative impact on calcium and phosphorus metabolism of Qianbei-Pockmarked goats in the area. To prevent phosphorus deficiency in livestock, oral supplements of bone meal, phosphate and mineral mixtures is recommended [6,30].

Some response criteria have been used to evaluate the phosphorus status of livestock, including serum values of phosphorus, calcium, and AKP [31]. A previous study suggested that bones are more sensitive to phosphorus than to other minerals. A marked hypophosphataemia is also a good indicator of severe phosphorus deficiencies, even when serum values of calcium are healthy. Phosphorus values in blood are not a good indicator of phosphorus status because phosphorus values can be normal for long periods after livestock have been exposed to serious phosphorus deficiencies [1,32].

Phosphorus deficiency disorders should be differentiated from chronic fluorosis in mature ruminants [6,33,34]. The typical characteristics of fluorine toxicity includes enlargements on the shafts of long bones, and mottling and pitting of teeth. In the present study, the fluorine contents of soil and herbage were lower than the critical values of 40 ppm [17,35,36]. Fluorine contents in bones, blood, and wool were within the healthy range. Therefore, the disorder in Qianbei-Pockmarked goats was not related to fluorosis. It is reasonable to conclude that the disorder among Qianbei-Pockmarked goats on the Yunnan-Guizhou Plateau, China is a locally nutritional and metabolic disease caused by low phosphorus in forage.

## Figures and Tables

**Table 1:** Results of clinical examination of affected Qianbei-Pockmarked goats

Characteristic	Male (10)1)	Non-pregnant (10)1)	Pregnant (10)1)	Ante-partum (10)1)	Post-partum (10)1)	Lamb (10)1)
Incidence (%)	0.00^a^	10.00	30.00	40.00	40.00	50.00
Mortality (%)	0.00^a^	0.00	33.33	50.00	50.00	60.00
Temperature (°C)	37.11±1.13	37.92±1.17	37.57±1.11	38.17±1.15	37.36±1.19	37.76±1.19
Heart rate (beats/min)	57.31±7.96	58.63±9.67	59.37±8.75	59.69±9.26	59.18±7.93	59.98±7.93
Respiratory rate (breaths/min)	17.39±2.73	17.32±2.52	18.59±2.59	19.19±2.74	19.33±2.37	22.33±2.37

1) Number of samples.

a Results between male and other goats were significantly different (p<0.01).

**Table 2:** Biochemical parameters in Qianbei-Pockmarked goats

Blood parameters	Affected animals	Healthy animals
LDH (μmoL/L)	5.76±1.38^a^	3.67±0.57
γ-GT (IU/L)	24.39±3.78	25.27±3.51
AKP (IU/L)	111.76±17.33^a^	53.28±8.76
CRT (μmoL/L)	143.56±35.67^a^	109.74±21.22
Ca (mmoL/L)	2.47±0.21	2.56±0.23
IP (mmoL/L)	1.25±0.22^a^	2.49±0.21

LDH, lactate dehydrogenase; AKP, alkaline phosphatase; γ-GT, γ-glutamyl transferase; CRT, creatinine; Ca, calcium; IP, inorganic phosphorus.

a Results between affected and healthy Qianbei-Pockmarked goats were significantly different (p<0.01).

**Table 3:** Serum protein contents in Qianbei-Pockmarked goats

Protein parameters	Affected animals	Healthy animals
Total protein (g/L)	63.63±4.22	65.36±4.32
Albumin (g/L)	45.15±3.77	45.61±3.79
α-Globulin (g/L)	3.82±0.51^a^	2.97±0.92
β-Globulin (g/L)	4.83±0.62^a^	3.17±0.81
γ-Globulin (g/L)	9.83±0.73^a^	13.61±1.21
A/G	2.44±0.51	2.31±0.37

A/G, albumin/globulin.

a Results between affected and healthy Qianbei-Pockmarked goats were significantly different (p<0.01).

**Table 4:** Mineral element contents in soil and forage samples

Elements	Soil		Forage	
	
	Affected	Healthy	Affected	Healthy
Cu (mg/kg)	17.73±2.56	17.92±2.53	6.59±2.37	6.36±2.79
Mo (mg/kg)	1.13±0.27	1.18±0.28	1.15±0.17	1.17±0.18
Fe (mg/kg)	4,227±321	4,312±313	356±37	362±35
Zn (mg/kg)	22.71±4.35	23.23±4.17	5.47±1.22	5.27±1.78
Mn (mg/kg)	53.76±11.55	53.86±11.26	11.27±2.78	11.32±3.55
Ca (mg/kg)	12,278±457	12,719±419	2,866±217	2,783±192
P (mg/kg)	37.97±6.561	79.57±5.78	221±12^a^	1,329±37
F (mg/kg)	22.76±3.8	22.23±3.3	22.57±4.78	21.59±5.37
Ca:P	539:1	159:1	12.97:1	2.09:1

a Results between affected and healthy pastures were significantly different (p<0.01).

**Table 5:** Mineral element contents in blood and wool of Qianbei-Pockmarked goats

Elements	Blood		Wool	
	
	Affected	Healthy	Affected	Healthy
Cu (mg/kg)	0.77±0.21	0.75±0.23	5.13±1.21	5.19±1.11
Mo (mg/kg)	0.37±0.05	0.35±0.02	0.37±0.06	0.35±0.03
Fe (mg/kg)	532±22	533±23	353±25	336±15
Zn (mg/kg)	14.37±2.86	14.78±2.72	85.57±4.81	86.39±3.78
Mn (mg/kg)	0.57±0.18	0.56±0.15	5.37±1.26	5.33±1.27
Ca (mg/kg)	128±11	129±12	1,119±31	1,197±57
P (mg/kg)	243±24^a^	387±31	62.97±11.75^a^	97.71±13.76
F (mg/kg)	17.76±5.37	19.27±3.17	18.69±4.78	19.89±6.79

a Results between affected and healthy Qianbei-Pockmarked goats were significantly different (p<0.01).

**Table 6:** Mineral element contents in bones and teeth of Qianbei-Pockmarked goats

Elements	Rib		Hip		Teeth	
		
	Affected	Healthy	Affected	Healthy	Affected	Healthy
Cu (mg/kg)	7.73±2.29	7.68±2.39	5.57±1.36	5.63±1.31	4.72±0.75	4.77±0.74
Mo (mg/kg)	1.27±0.31	1.25±0.31	2.67±0.56	2.63±0.51	2.37±0.27	2.38±0.33
Fe (mg/kg)	178±11	177±12	176±18	165±14	159±11	155±12
Zn (mg/kg)	123±11	122±12	97.38±6.81	97.59±6.39	91.7±5.78	91.89±5.27
Mn (mg/kg)	6.57±1.37	6.61±1.26	4.33±1.27	4.37±1.15	6.19±0.59	6.18±0.61
Ca (g/kg)	139±12	138±12	129±17	128±23	176±24	175±25
P (g/kg)	36.38±5.35^a^	74.78±11.69	34.76±3.18^a^	75.38±11.11	35.33±5.17^a^	76.66±7.38
F (mg/kg)	55.37±8.75	56.17±7.68	64.46±8.73	64.37±8.67	76.36±9.17	77.69±7.37

a Results between affected and healthy Qianbei-Pockmarked goats were significantly different (p<0.01).

**Table 7:** Mineral element contents in blood in treatment and control groups

Elements	Treated animals			Control animals		
	
	0 d	10 d	20 d	0 d	10 d	20 d
Cu (mg/kg)	0.75±0.15	0.74±0.19	0.73±0.13	0.75±0.16	0.73±0.14	0.74±0.15
Mo (mg/kg)	0.33±0.03	0.32±0.05	0.34±0.06	0.35±0.02	0.33±0.03	0.34±0.04
Fe (mg/kg)	539±21	537±25	533±27	533±23	532±22	529±22
Zn (mg/kg)	15.27±2.26	14.89±2.56	15.12±2.36	15.76±2.72	14.99±2.16	15.37±2.26
Mn (mg/kg)	0.59±0.17	0.57±0.18	0.56±0.12	0.55±0.15	0.58±0.15	0.57±0.13
Ca (mg/kg)	123±12	128±11	125±13	129±15	127±14	126±13
P (mg/kg)	247±22^a^	393±37^a^	433±32^a^	253±21	249±23	248±29
F(mg/kg)	17.16±2.37	17.76±3.36	17.36±3.32	18.12±3.17	17.86±3.31	17.97±3.37

a Significant differences (p<0.01) in the treated group.
